# Genome-wide characterization and functional identification of *MYB* genes in *Malus sieversii* infected by *Valsa mali*

**DOI:** 10.3389/fpls.2023.1112681

**Published:** 2023-04-05

**Authors:** Yu Ding, Qihang Yang, Abdul Waheed, Mingqi Zhao, Xiaojie Liu, Gulnaz Kahar, Yakupjan Haxim, Xuejing Wen, Daoyuan Zhang

**Affiliations:** ^1^ State Key Laboratory of Desert and Oasis Ecology, Key Laboratory of Ecological Safety and Sustainable Development in Arid Lands, Xinjiang Institute of Ecology and Geography, Chinese Academy of Sciences, Urumqi, China; ^2^ College of Resources and Environment, University of Chinese Academy of Sciences, Beijing, China; ^3^ Xinjiang Key Laboratory of Conservation and Utilization of Plant Gene Resources, Xinjiang Institute of Ecology and Geography, Chinese Academy of Sciences, Urumqi, China; ^4^ Turpan Eremophytes Botanical Garden, Chinese Academy of Sciences, Turpan, China

**Keywords:** MsMYB transcription factor, *Malus sieversii*, Valsa canker, fungal resistance, function identification

## Abstract

Among the most important transcription factors in plants, the v-myb avian myeloblastosis viral oncogene homolog (*MYB*) regulates the expression network of response genes under stresses such as fungal infection. In China, the canker disease *Valsa mali* threatens the survival of *Malus sieversii*, an ancestor of cultivated apples. Using the *M. sieversii* genome, we identified 457 *MsMYB* and 128 *R2R3-MsMYB* genes that were randomly distributed across 17 chromosomes. Based on protein sequence and structure, the *R2R3-MsMYB* genes were phylogenetically divided into 29 categories, and 26 conserved motifs were identified. We further predicted *cis*-elements in the 2000-kb promoter region of *R2R3-MsMYB*s based on the genome. Transcriptome analysis of *M*. *sieversii* under *V. mali* infection showed that 27 *R2R3-MsMYBs* were significantly differentially expressed, indicating their key role in the response to *V. mali* infection. Using transient transformation, *MsMYB14*, *MsMYB24*, *MsMYB39*, *MsMYB78*, and *MsMYB108*, which were strongly induced by *V. mali* infection, were functionally identified. Among the five *MsMYBs*, *MsMYB14* and *MsMYB78* were both important in enhancing resistance to diseases, whereas *MsMYB24* inhibited resistance. Based on the results of this study, we gained a better understanding of the *MsMYB* transcription factor family and laid the foundation for a future research program on disease prevention strategies in *M. sieversii*.

## 1 Introduction

Plants rely on a wide range of transcription factors (TFs), including the v-myb avian myeloblastosis viral oncogene homolog (*MYB*) TFs, to respond to biotic stress and other environmental factors (Romero et al., 1998; Riechmann et al., 2000). In 1987, the first *MYB* gene, *COLORED1* (*C1*), was identified in plants (Paz-Ares et al., 1987). The *MYB* repeats (R) that form the *MYB* DNA-binding domain (DBD) comprise four typical subfamilies and atypical *MYB*-like subfamilies (Mmadi et al., 2017).

*MYB* TFs have a modular structure, with an N-terminal domain called the MYB domain and a C-terminal that could either be activator, repressor, or both (Stracke et al., 2001). Several encoded proteins have highly conserved domains composed of 1-4 imperfect amino acid sequence repeats (R) of 50–53 amino acids arranged in a helix-turn-helix structure (HTH) (Ogata et al., 1996; Rosinski and Atchley, 1998; Dubos et al., 2010; Hurtado-Gaitán et al., 2021). In the three-helical structure, there is a hydrophobic core formed by three tryptophans positioned periodically (Ogata et al., 1992; Dubos et al., 2010; Zhang et al., 2012). In accordance with the number of repeats, four main subfamilies were classified: R1 or R2-MYB (1R), R2R3-MYB (2R), R1R2R3-MYB (3R), and R4-MYB (4R), with one, two, three, and four conservative *MYB* repeats, respectively (Kanei-Ishii et al., 1990). The R2R3-MYB subfamily is considered the largest of the MYB family (Sun et al., 2019; Chen X. et al., 2021). Furthermore, R1R2R3-MYB has been reported to exist predominantly in land plants (Kranz et al., 2000). The most remarkable is the 4R-MYB group, which includes four R1/R2-like repeats (Dubos et al., 2010). Because of these DBD characteristics, the R2R3-MYB family was also divided into 25 subgroups (SGs) in *Arabidopsis* (Stracke et al., 2001). In recent years, genome-wide sequencing has been performed in various species of plants, and *MYB* genes were discovered in many species (Pucker et al., 2020; Chen G. et al., 2021; Chen L. et al., 2021; Song et al., 2021). Moreover, *MYB* genes have been found to function in several regulatory mechanisms and simultaneously perform multiple functions. *MYB* genes were also found to be expressed in different organs and tissues by expression profiling, and several genes were classified as being induced by external stress conditions (Li X. et al., 2019; Xing et al., 2021; Yuan et al., 2021). Moreover, *MYB* genes are also responsible for disease resistance in many plants, including sugarcane (*Saccharum officinarum*) (Yuan et al., 2021). In addition to improving product quality and yield, screening *MYB* genes can increase bioenergy resources in biotechnology (Cardenas-Hernandez et al., 2021).

Apples are very significant fruit crops (Dubos et al., 2010) and prominent economic trees. Infection of apple tree trunks, scaffolds, branches, and leaves by diseases such as Valsa canker and Botryosphaeria canker causes huge destruction to production (Li et al., 2021; Liang et al., 2022). In particular, the ascomycete *V. mali* has been reported to greatly reduce apple production (Lee et al., 2006; Wang et al., 2014). Recent research suggests that *MYB* plays a significant role in controlling apple canker. MYB TFs are known to play a crucial role in stress perception and signal transduction, especially in the stress response in plants (Yanhui et al., 2006; Lu et al., 2009; Li J. et al., 2019). The balance between homeostasis and the environment is maintained by several genes involved in hormone regulation (Chen X. et al., 2021). In recent years, many differentially expressed genes that participate in biotic and abiotic stress responses have been identified in plants by transcriptomic analysis. In recent years, R2R3-MYB proteins perform a variety of functions under biotic and abiotic stress, including secondary metabolic regulation and the cell cycle, growth, and development (Paz-Ares et al., 1987; IH and EP, 1999; Stracke et al., 2001; Dubos et al., 2010; Blanco et al., 2018; Chen et al., 2019; Cao et al., 2020; Pucker et al., 2020). Several studies have shown that R2R3-MYB proteins regulate anthocyanin and lignin biosynthesis in plants (Tuan et al., 2015; Arce-Rodriguez et al., 2021; Song et al., 2021; Zhou et al., 2021). As a result of *R2R3-MYB* regulation, anthocyanin biosynthesis is upregulated through hypomethylation of DNA in their promoter regions (Zhou et al., 2021). A certain level of canker resistance can be indirectly enhanced by the growth of anthocyanins and lignin (Faize et al., 2020; Zhang Q. et al., 2021). Overexpression of *R2R3-MYB* genes as a method for preventing phytopathogenic fungi such as *V. mali* and *B. kuwatsukai* is effective for protecting plants from phytopathogenic fungi (Chen L. et al., 2021).

Herbaceous and woody plants have been studied for the majority of the functions and structural characteristics of the *MYB* gene family. Several studies have shown that MYB TFs are involved in regulating plant growth and development in wild apples (*Malus sieversii*) and in modulating cold tolerance (An et al., 2018; Xie et al., 2018). Many R2R3-MYB proteins in *M. sieversii* cannot be accurately identified as redundant in their respective functions, but they probably have overlapped functions (Jin and Martin, 1999). In this study, the resistance functions of five R2R3-MYB TF in Valsa canker were identified. As a first step, this research collected genome-wide information about *M. sieversii* and snapped all *MYB* gene sequences from *A. thaliana*. These analyses were conducted to predict protein physicochemical properties, build a phylogenetic tree, analyze conserved motifs and chromosomal positions, and forecast promoter *cis*-elements. Five *MsMYB* transcripts (*MsMYB14*, *MsMYB24*, *MsMYB39*, *MsMYB78*, and *MsMYB108*) were cloned to determine their resistance to Valsa canker by analyzing the transcriptome data of the *MYB* gene. This study helps to understand the mechanisms of *MYB* genes and to identify *V. mali*-resistant genes.

## 2 Materials and methods

### 2.1 Identification and classification of the *MYB* gene family in *Malus sieversii*

In this study, the Hidden Markov model (HMM) was used to identify *MsMYB* based on the *M. sieversii* genome, which was retrieved from the Pfam database (PF00249) (http://pfam.xfam.org/). Additionally, AtMYB protein sequences were retrieved from TAIR (http://www.arabidopsis.org/) (Stracke et al., 2001) and used for searching more MYB transcription factor candidates using BlastP and E-value < 1e^-5^.

To predict the molecular weight (Da) and isoelectric point (PI) of potential proteins, the ProtParam tool was used (https://web.expasy.org/protparam/). TBtools were used to measure length of the *MsMYB* gene and MsMYB protein sequences (Chen et al., 2020) (**Supplementary Table S1**).

### 2.2 Phylogenetic and conserved motif analysis of the *MYB* gene family

To identify and classify 1R, 2R, 3R, and 4R subfamilies of MYB and thoroughly classify the R2R3-MYB subfamily, AtMYB and MsMYB were used to analyze multiple sequence alignments and construct a phylogenetic tree with MEGA 10 using the neighbor-joining (NJ) model and 1000 replicate bootstraps. Finally, a phylogenetic tree was constructed using itol (https://itol.embl.de/).

To evaluate the classification results of MsMYB, MEME, an online analysis tool (https://meme-suite.org/meme/doc/meme.html), was used to analyze and show the MYB domain structure. The parameters included motif occurrence distribution, zero or one motif per sequence, the maximum number of motifs (26), and the optimal width of motifs between 6 and 10 residues.

### 2.3 Analysis of gene collinearity and *cis*-elements of promoter

The *Malus domestica* Borkh genome was downloaded from the GDR database (https://www.rosaceae.org/species/malus/all) and used for comparative analysis (Daccord et al., 2017). To explore the collinearity relationship of the *MsMYB* gene family, the McScan module in TBtools was used to analyze collinearity of intragenome and intergenome, and Advance Circos and Multiple Synteny Plot were used to perform the collinearity analysis and duplication type, including the whole genome duplication or segmental (WGD or segmental), tandem, Dispersed and Proximal. Based on the genome, the chromosomal distribution of *R2R3-MsMYB* was also shown using TBtools. To determine the type and number of *cis*-elements of the *R2R3-MsMYB* gene, the promoter region, upstream 2000 bp of the gene was extracted from the genome for analysis. We submitted the promoter sequences to the PlantCARE database (https://bioinformatics.psb.ugent.be/webtools/plantcare/html/) for excavating *cis*-elements.

### 2.4 Expression profiling of *MsMYB* genes in response to *Valsa mali*

A set of transcriptions of *M. sieversii* under *V*. *mali* infection data was downloaded from NCBI for analysis of *R2R3-MsMYB* gene expression patterns (https://www.ncbi.nlm.nih.gov/) (Liu et al., 2021). A heat map of *R2R3-MsMYB* gene expression was created using the TBtools software. Additionally, a gene with a differential expression scale of Log_2_|foldchange| ≥ 1 and p-value < 0.05, i.e., a differentially expressed gene (DEG) under infection, was compared to the control (**Supplementary Table S3**).

### 2.5 Experimental materials and gene cloning

*Malus sieversii* seedlings were grown under controlled conditions in a greenhouse with soil (nutrient soil: vermiculite: perlite = 3:1:1). They were kept growing until they were 2–3 months old, at 24–26°C, 70%–75% relative humidity, and sufficient watering.

The intact coding sequence (CDS) of the examined TF was fused in-frame with the C-terminus of a 3 × Flag tag under the control of the *CaMV 35S* promoter in the *p1307-Flag* plant expression vector. All primers used for construction are listed in **Supplementary Table S4**. DNA sequencing was performed on all constructs before transferring them to *Agrobacterium EHA105*.

### 2.6 Identification of the resistance of MsMYB transcription factors in response to *Valsa mali*

Transient transformation was performed as described by Wen et al. (2020). The colonies of *Agrobacterium tumefaciens* EHA105 harboring the studied constructs were cultured in LB medium to OD 08-1.0, and harvested by centrifugation at 3500 rpm. *Agrobacterium tumefaciens EHA105* colonies harboring the studied constructs were adjusted to an OD600 of 50 mL of transformation solution [ 5% (w/v) sucrose + 150 μM acetosyringone + 5 mM CaCl_2_ + 0.015% DTT (w/v) + 20 μM 5-AZA + Tween 20 (0.01%, v/v), pH 5.8]. *Malus sieversii* leaves were soaked in the transformation solution at room temperature while shaking at 100 rpm. Three hours after the leaves were removed from the transformation solution, excess water was quickly wiped off with sterile filter paper. The leaves were rinsed twice with sterile water. Control plants (Con) were transformed with empty *P1307-Flag*.

Aseptically, mycelial plugs (diameter of 0.5 mm) were removed from the edge of the three-day-cultured isolate *V. mali* on PDA medium. Following transient expression, the leaves were wounded by tips and inoculated with mycelial plugs for 24 h. For three days, the inoculated leaves were placed on dishes tapped with parafilm to maintain moisture. Incidence ratios (%) were calculated using photographs. We measured the lesion areas using ImageJ software. Three biological replicates were used for each experiment. Using the LSD method, the mean and standard error (SD) of the data were analyzed using one-way ANOVA (p < 0.05). For the measurement of fungal biomass, we used the following equation:6.02 × 10 ^ 23 × (ng/μl × 10 ^ −9) × (DNA length × 660) × volume of extraction liquid ÷ fresh weight of sample; for measuring H_2_O_2_ and MDA content, we used the H_2_O_2_ content detection kit (Jiancheng, Nanjing) and the MDA content detection kit (Jiancheng, Nanjing), respectively.

### 2.7 Real-time PCR analysis

*Malus sieversii* RNA was isolated using the CTAB method, and DNA contamination was removed with DNase I. Using the PrimeScript TMRT reagent kit (Transgen, Beijing, China) with oligo (dT) primers, 1 µg of total RNA from each sample was reverse-transcribed into cDNA. The resulting cDNA product was diluted 10-fold before use in the PCR. To normalize the number of templates generated, *EF1αgene* (*Elongation factor 1-α*) was used as the internal reference.

A CFX96 Real-Time PCR Detection System (Bio-Rad, CFX96, USA) was used to perform real-time PCR under the following conditions: 94°C for 60 s; 45 cycles at 94°C for 10 s, 59°C for 20 s, 72°C for 30 s, and 80°C for 1 s for plate reading. In a 20-L volume, the reaction mixture included 10 L of SYBR Green Real-time PCR Master Mix (Transgenic, Beijing), 0.5 L of forward and reverse primers, and 2 L of cDNA template. A melting curve was generated to ensure the purity of the amplified products. Three biological replicates were used in all experiments, and relative expression levels were assessed using the 2^−ΔΔCt^ method (Livak and Schmittgen, 2001). Detailed primer information is provided in **Supplementary Table S4**.

## 3 Results

### 3.1 Genome-wide identification of the *MYB* gene family in *Malus sieversii*

The Hemmer search that based PF00249 model and BlastP that use ATMYB protein sequences as query were used to identify MsMYB TFs in *M. sieversii*. When 458 proteins were analyzed for R1-4 motifs, one protein without any conserved motifs was found. Finally, the 457 proteins were identified. Based on conserved motifs and phylogenetic analysis, we identified 323, 128, 5, and 1 member in the 1R, 2R(R2R3), 3R, and 4R subfamilies, respectively. The 1R-MsMYB subfamily was divided into 52 groups. The isoelectric point (PI) of R1-MsMYB proteins ranged from 4.38 for MS12G07430.1 to 10.24 for MS04G02770.1. The 2R-MsMYB subfamily was divided into 29 groups. The PI of R2R3-MsMYB proteins ranged from 4.83 for MS03G17360.1 to 10.43 for MS13G03930.1, and 56% of the proteins had isoelectric points between 5 and 7. The average PI of the 2R-MsMYB subfamily was 7.05 (STD = 0.13). The maximum and minimum molecular weights were 259.85 kDa (MS03G19210.1) and 6.76 kDa (MS10G15090.1), respectively. The average gene length of each subfamily was arranged in the following order: 2R (992 bp), 1R (1303 bp), 3R (2030 bp), and 4R (2928 bp) (**Supplementary Table S1**).

### 3.2 Phylogenetic and motif analysis of R2R3-MsMYB transcription factors

**Figure 1:**
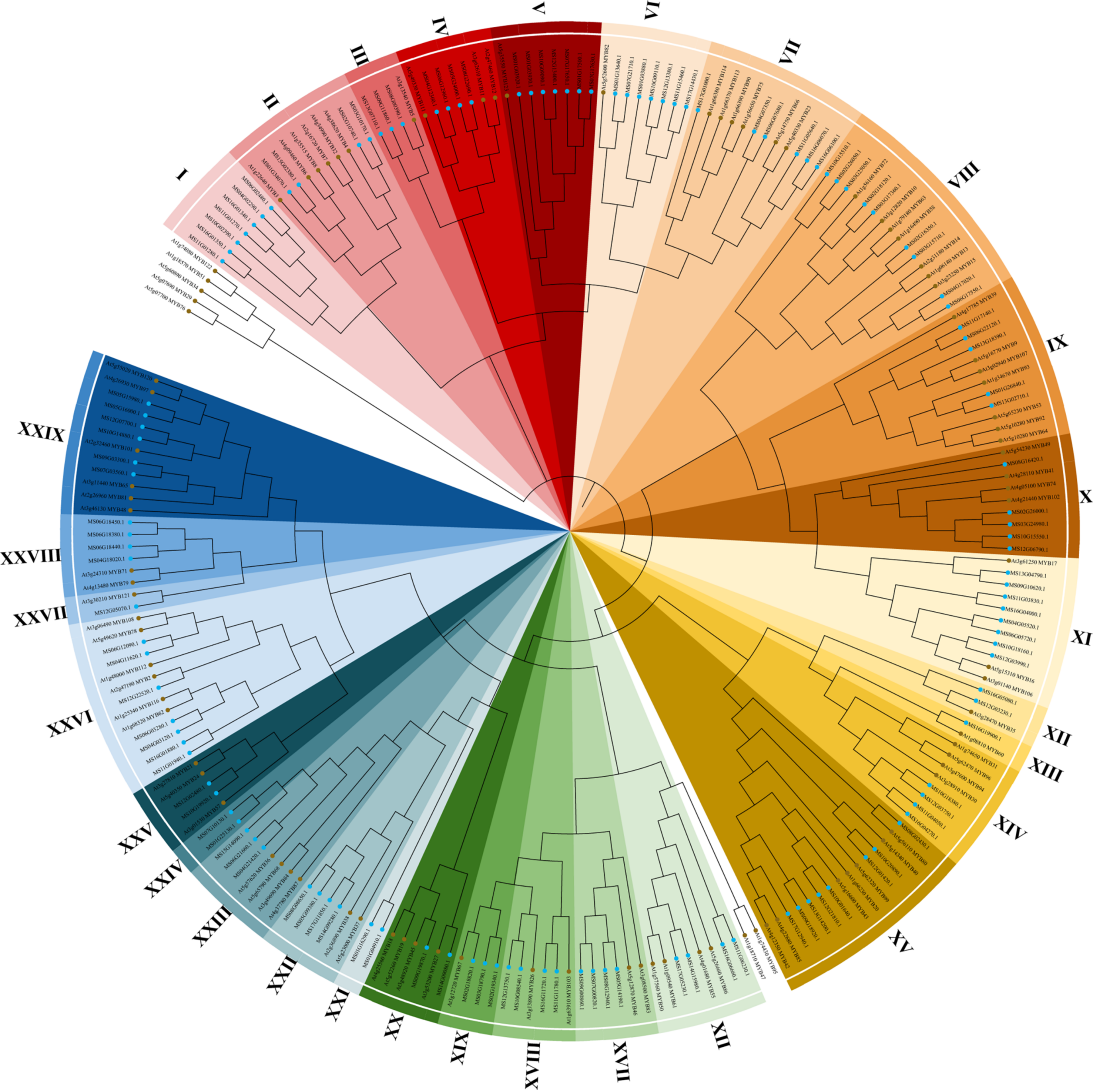
Phylogenetic analysis of R2R3-MYB transcription factors from *Malus sieversii* and *Arabidopsis thaliana*. Neighbor-joining tree of 128 *M. sieversii* (MsMYBs) and 94 *A. thaliana* (AtMYBs) R2R3-MYB TFs. The bootstrap test used 1000 replicates. The R2R3-MYB transcription factors of *M. sieversii* and *A. thaliana* are marked in blue and brown, respectively. The tree shows 29 clades (I–XXIX) with a high bootstrap value (with a different color for each clade). The uncolored region represents *A. thaliana* (AtMYBs).

The 2R subfamily was further classified and studied for diverse and important biological functions. The 94 R2R3-AtMYB and 128 R2R3-MsMYB that obtained the R2R3 domain were used to construct a phylogenetic tree and analyze conserved motifs. Finally, R2R3-MsMYBs were classified into 29 groups and named I-XXIX, and MsMYBs belonging to the same cluster had the same motif pattern (**Figure 1**).

**Figure 2:**
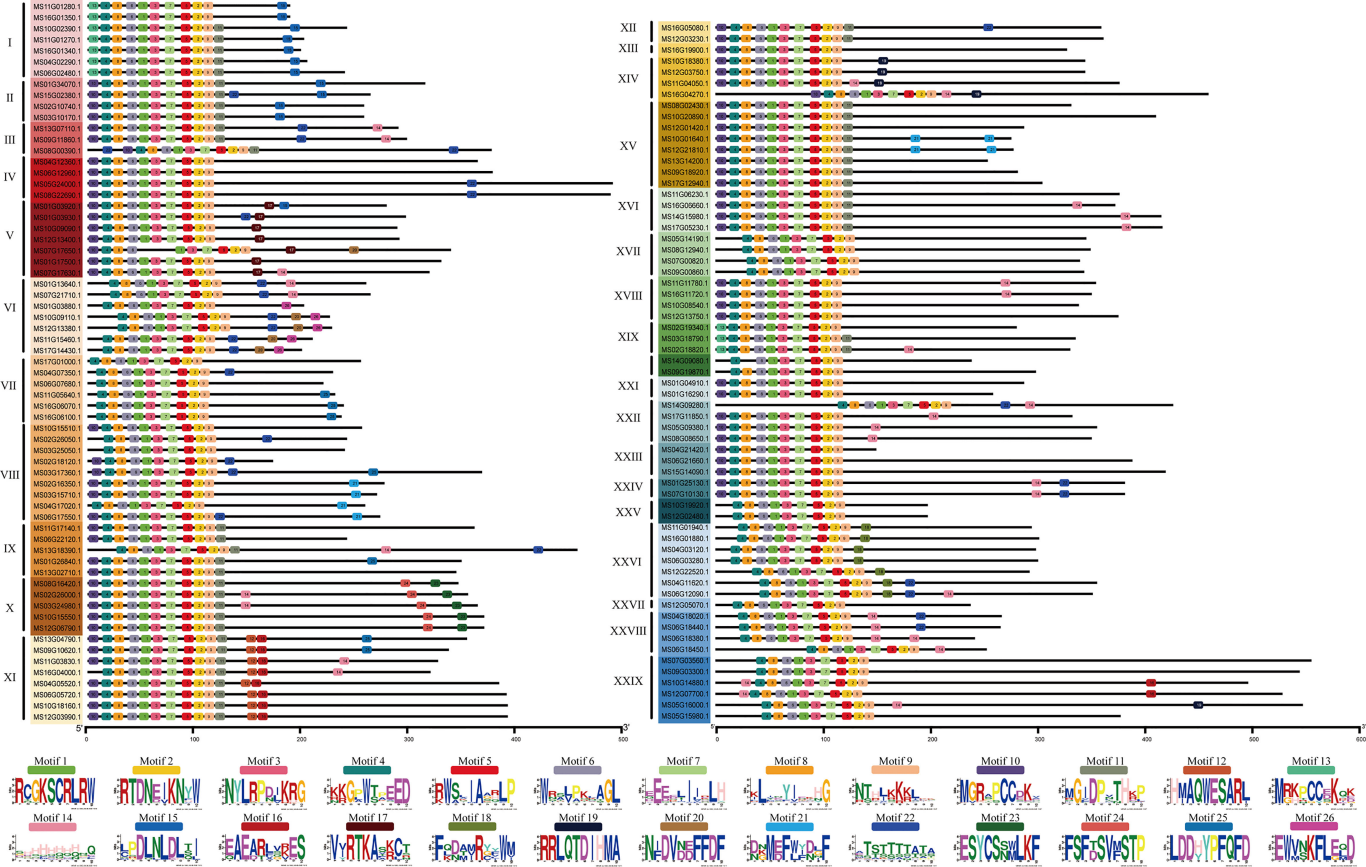
Motif composition of R2R3-MYBs in *Malus sieversii*. Roman numbers and different colors indicate different groups. Each solid line length indicate the different protein sequences length. The 9 Colored boxes indicate different motifs, which composes the MYB conserved domain region.

To further verify the 128 R2R3-MsMYB classification results by phylogenetic analysis, the MEME website was used to study the MYB domain structure. A total of 26 conserved motifs were identified in 128 R2R3-MsMYB proteins. Nine motifs, i.e., 1, 2, 3, 4, 5, 6, 7, 8, and 9, were highly conserved in the subfamily and constructed a basal MYB domain of 50–53 amino acids (**Figure 2**). Among them, motifs 4, 8, 6, 1, 3, and the first half of motif 7 formed a complete R2 domain, whereas the second half of motif 7 and motifs 5, 2, and 9 formed a complete R3 domain. In addition, MsR2R3-MYB members within the same clade shared similar motifs and highly conserved MYB domains. Among these, motif 17 formed a common element in the V group. Groups I and II had a common motif 15; the X group had unique motifs, including motifs 23 and 24 in R2R3-MYB TF; motifs 12 and 16 were unique to the XI group; motif 19 was unique to the XIV group; and motif 18 was unique to XXVI. Specifically, motif 21 was repeated twice in MS10G01640.1 and MS12G21810.1, but repeated once in MS02G16350.1, MS03G15710.1, MS04G17020.1, and MS06G17550.1. MS16G1880.1 (MsMYB24), MS06G12090.1 (MsMYB78), and MS06G12090.1 (MsMYB108) were clustered in group XXVI (**Figure 2**). A phylogenetic tree is generally built based on protein structures and conservation domains that are characteristic of members of the subfamily. Phylogenetic trees can be verified for their accuracy and authenticity based on these characteristics.

### 3.3 Chromosomal distribution and gene collinearity of MsMYB genes

**Figure 3:**
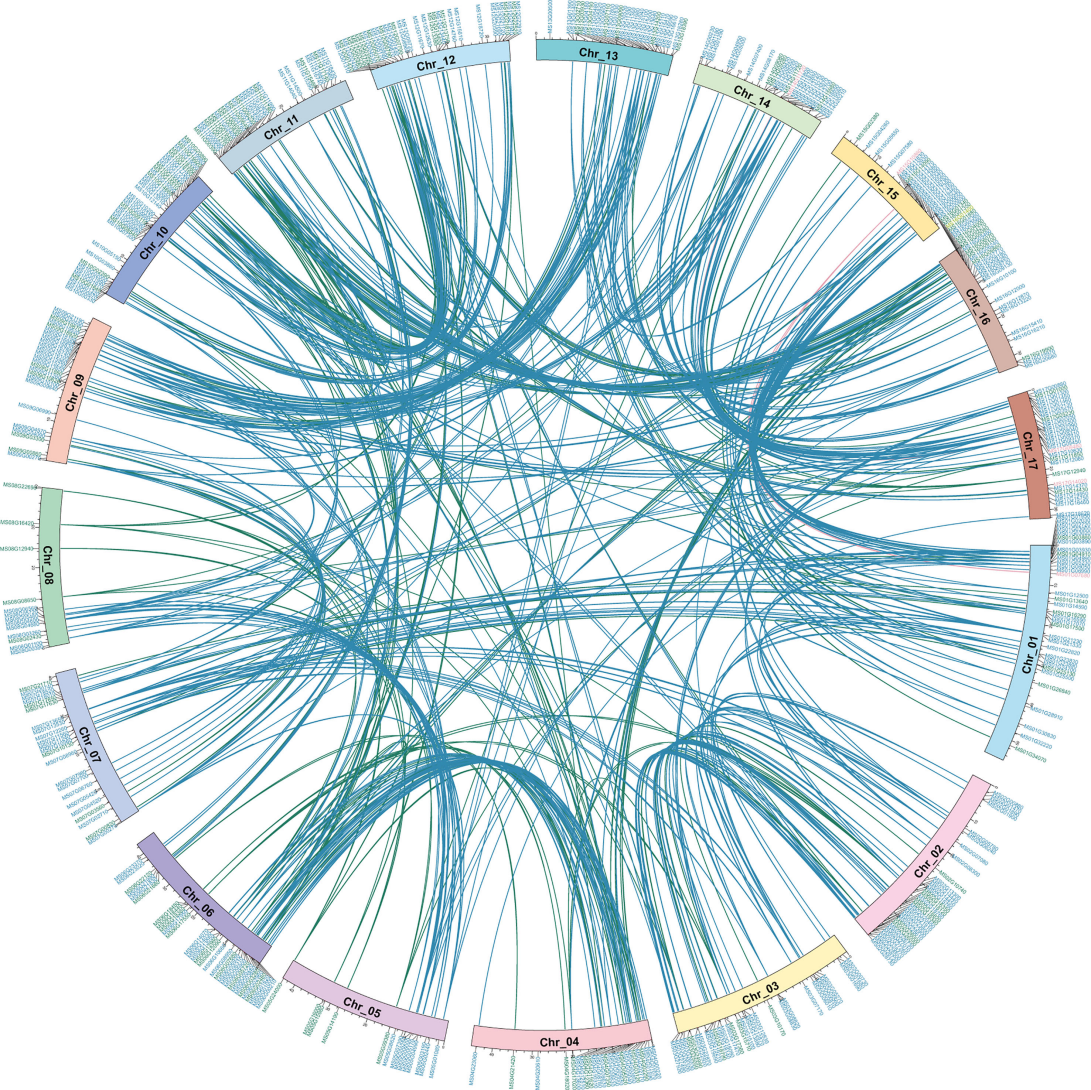
Self-Collinearity analysis and distribution of *MsMYB* genes in the *Malus sieversii* genome. Circle plot shows collinearity and distribution of the *MYB* gene family, and the colored lines highlightly link two syntenic *MYB* genes. Colored lines indicate collinearity relationship of *1R-MsMYB* (blue), *R2R3-MsMYB* (green), and *3R-MsMYB* (pink). Location of *MYB* genes on apple chromosomes (Chr_01~17). Colored IDs indicate *1R-MsMYB* (blue), *R2R3-MsMYB* (green), *3R-MsMYB* (pink), and *4R-MsMYB* (yellow), respectively.

To clearly show the distribution and better understand the origin of the *MsMYB* gene family, McScan was used to analyze collinearity relationships in self-*MsMYB* genes and between *M. sieversii* and *M. domestica*. The 457 *MsMYB* genes distributed across 17 chromosomes did not cluster as a subfamily. Based on the *R2R3-MsMYB*, a total of 13 *MYB* genes were found on chromosome 12, which contained the most genes, followed by 12 genes on chromosome 10 and 12 genes on chromosome 6. In contrast, only two genes were identified on chromosome 15: *MS15G02380* and *MS14G15980*. The *1R-MsMYB* subfamily with the largest genes and *R2R3-MsMYB* uneven are present on each chromosome. Five genes were identified in the 3R subfamily. In addition, two *3R-MsMYB* genes were distributed on the same chromosome, whereas the other three were distributed on different chromosomes. Meanwhile, 452 link gene pairs were produced, including 323 *1R-MYB* genes, 128 *R2R3-MYB* genes, and one *3R-MYB* gene. Among *1R-MYB* genes, they mainly matched *1R-MYB* (88.75%), whereas 11.25% *1R-MYBs* were matched with the *R2R3-MYB* genes. In *R2R3-MYB* subfamily, there were 84.72% self-matches and 15.28% others-matches, including 13.89% *1R-MYB* subfamily and 1.39% *3R-MYB* subfamily (**Figure 3**). The collinearity result showed that for 457 *MsMYB* genes, 457 duplication events occurred, including 392 WGD or segmental, 45 dispersed, 10 tandem, seven proximal, and three singleton events. The results indicated that WGD or segmental was the main amplification driving force in *MsMYB* gene family formation process (**Supplementary Table S2**).

**Figure 4:**
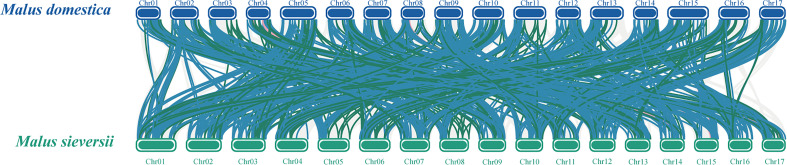
Collinearity analysis of *MYB* genes between *Malus domestica* and *Malus sieversii*. Syntenic regions between *Malus domestica* and *Malus sieversii* chromosomes are connected by different color lines. Gray lines and colored lines represent the collinear blocks and syntenic *MYB* gene pairs, respectively, including *1R-MYB* (blue), *R2R3-MYB* (green), and *3R-MYB* (pink).

To better reveal the evolution and function of the *MYB* gene, we performed a collinearity analysis between *M. sieversii* and *M. domestica*. In total, 413 *MYB* collinearity gene pairs were obtained between the genomes of *M. sieversii* and *M. domestica*. The *MsMYB* gene family contained 286 *1R-MYB*, 122 *R2R3-MYB*, 4 *3R-MYB*, and 1 *4R-MYB* collinear gene pairs. In addition, 44 *MsMYB* genes did not correspond to *M. domestica* genes, including 37 *1R-MsMYBs* and seven *R2R3-MsMYBs*, which indicates that those *MsMYB* genes were lost during evolution (**Figure 4**).

### 3.4 Identifying *cis*-elements in *R2R3-MsMYB* promoters

**Figure 5:**
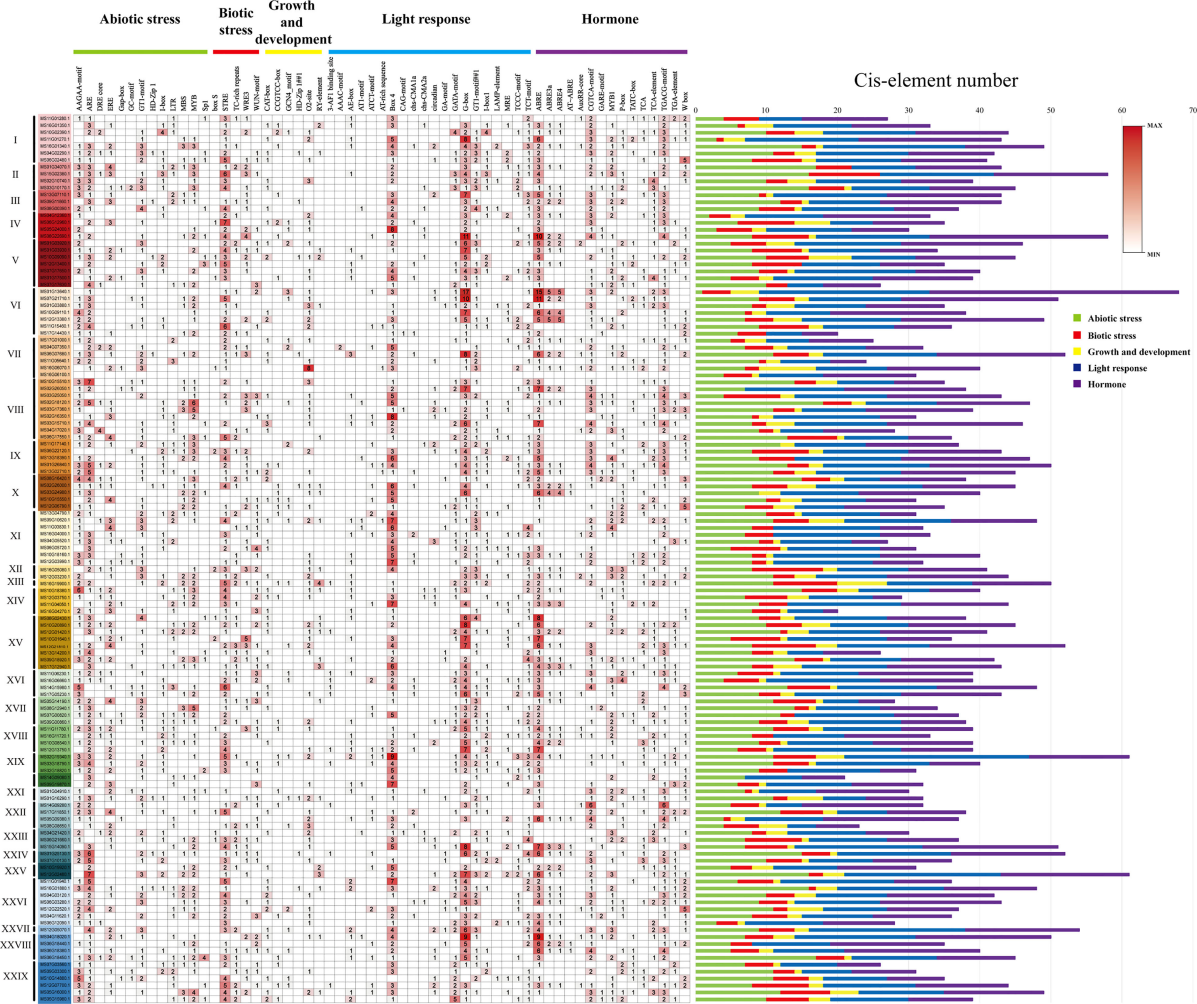
Analysis of the *cis*-acting elements in the promoter region of the *R2R3-MsMYB* genes. Top: different color bars indicate the classification of different *cis*-elements. I–XXIX show the subfamily of *R2R3-MsMYB* genes.

The *cis*-elements in the promoter region of 128 *R2R3-MsMYB* genes were analyzed to better understand the functions of *MsR2R3-MYBs* in wild apple plants. A total of 59 *cis*-acting elements were identified, including 13 abiotic stress elements, five biotic stress elements, six growth development-related elements, 20 light-response elements, and 15 hormone response elements (**Figure 5**). Among them, the maximum number of light-response elements, especially Box 4 (114/128, 89%) and G-box elements (111/128, 86%), was followed by 87 GT1-motifs (68%), 85 TCT-motifs (66%), among others. Among the growth- and development-related elements, only one was greater than 50%, namely the O2-site (65/128, 50%), followed by 54 CAT-boxes (42%), 26 CCGTCC-boxes (20%), 25 GCN4_motif (19%), 24 RY-elements (18%), and 8 HD-Zip1 (6.2%). Among the hormone response elements, ABRE (113/128, 88%), GARE-motif (96/128, 75%), TGACG-motif (96/128, 75%), and MYB1 (81/128, 63%) were related to abscisic acid (ABA) and methylated jasmonic acid (MeJA), respectively. Among the biotic response elements, only one was greater than 80%, namely STRE (89/128, 83%), while the others were less than 50%. Among the abiotic stress elements, several higher numbers of *cis*-elements were found: ARE (110/128, 86%), AAGAA-motif (94/128, 73%), GT1-motif (87/128, 68%), and MYB (78/128, 61%). Promoter analysis showed that 55 of the 128 selected *MsMYB* promoters harbored the same number of CGTCA-motif (75%) and TGACG-motif (75%). It also showed that 55 of the 128 selected *MsMYB* genes harbored the same number of ABRE3a (43%) and ABRE4 (43%) *cis*-elements. Compared to *MsMYB14* and *MsMYB39*, *MsMYB24*, *MsMYB78*, and *MsMYB108* had the same number of *cis*-elements, ABRE3a and ABRE4, and they were in group XXVI. According to the distribution of biological stress response *cis*-elements, each gene had a common biotic stress response *cis*-elements STRE in group IV, and *MS06G12960.1* had the highest number of STRE, which was seven. Ultimately, *MsMYB24*, *MsMYB14*, *MsMYB39*, *MsMYB78*, and *MsMYB108* had one, seven, four, three and five biotic stress elements, respectively.

### 3.5 Expression patterns of *R2R3-MsMYB* genes upon the *Valsa mali* infection

**Figure 6:**
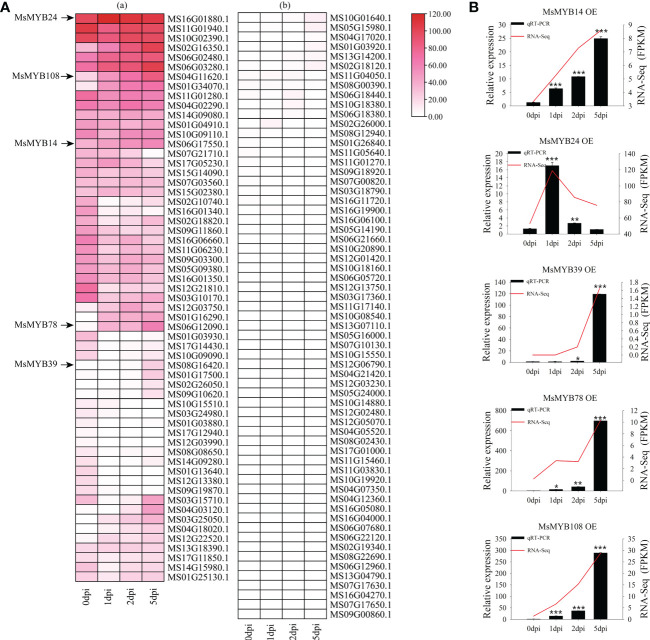
Expression profiles of the 128 *R2R3*-*MsMYB* genes during response to infection by *Valsa mali*. **(A)** Heatmaps with clusters represent the expression profiles of *R2R3*-*MsMYB* genes at 0, 1, 2, and 5 dpi. Color scale represents the normalized FPKM values. Red, pink, and white indicate high expression, low expression, and no expression, respectively. The cut-off of the differentially expressed *R2R3*-*MsMYB* transcripts islog2 (fold change)≥ 1 (Q-value < 0.05). **(B)** Comparison of RNA-seq data (red line) with qRT-PCR data (black column). The FPKM values from the RNA-seq are shown on the right y-axis. The relative expression levels are shown on the left y-axis. One asterisk indicates a significant difference between treatment and control plants (p ≤ 0.05). Two asterisks indicate an extremely significant difference between treatment and control plants (p ≤ 0.01). Three asterisks indicate an extremely significant difference between treatment and control plants (p ≤ 0.001).

We analyzed the transcript levels of *MsR2R3-MYB* genes in wild apple leaves at different time points (0, 1, 2, and 5 dpi). According to the expression pattern after infection with *V. mali* and GO annotation analysis, the expression pattern was divided into several modules (**Figure 6A**). **Figure 6A** (a) and **Figure 6A** (b) show high and low expression of these genes, respectively. A total of 27 *MsMYB* genes showed the highest changes in transcription levels, with significant differences. Compared with these *MsMYB* genes on day 0, five *MsMYB* genes showed up-regulated transcriptional levels and seven *MsMYB* genes showed down-regulated transcriptional levels on day 1. In addition, 11 *MsMYB* genes were down-regulated and five *MsMYB* genes were up-regulated on day 2. Furthermore, 11 *MsMYB* genes were down-regulated and 13 *MsMYB* genes were up-regulated on day 5. On days 1, 2, and 5, there were four sustained up-regulated genes, namely *MS06G02480.1*, *MS01G34070.1*, *MS12G03750.1*, and *MS01G16290.1*. At all time points, there were five sustained down-regulated genes: *MS16G01340.1*, *MS03G10170.1*, *MS12G21810.1*, *MS01G03930.1*, and *MS01G13640.1*. Of the remaining genes, *MS04G11620.1* (*MsMYB108*) was upregulated on day 2. *MS06G17550.1* (*MsMYB14*) and *MS06G12090.1* (*MsMYB78*) only showed up-regulated expression on day 5. *MS06G17550.1* (*MsMYB14*) only showed up-regulated expression on day 1.

**Figure 6B** shows that the expression profiles were accurate. The RNA-Seq (FPKM) results were consistent with the qRT-PCR results. According to the results of qRT-PCR, *MsMYB14* continued to be highly expressed at 0, 1, 2, and 5 days. Compared with day 0, *MsMYB14* expression levels were 3.9 times higher on day 1, 7.3 times higher on day 2, and 18.1 times higher on day 5. On day 1, *MsMYB24* expression levels were 12-fold higher compared with day 0. As shown in **Figure 6B**, it was up-regulated and subsequently down-regulated. *MsMYB39* was upregulated in the late stage. *MsMYB78* gene expression was up-regulated 10-fold and 31-fold on days 1 and 2, respectively, compared to day 0, and was then up-regulated 536-fold. *MsMYB108* expression levels were 10.1 times higher on day 1, 28.3 times higher on day 2, and 222.1 times higher on day 5.

### 3.6 Identification of *R2R3-MsMYB* members in *Malus sieversii* in response to *Valsa mali* infection

**Figure 7:**
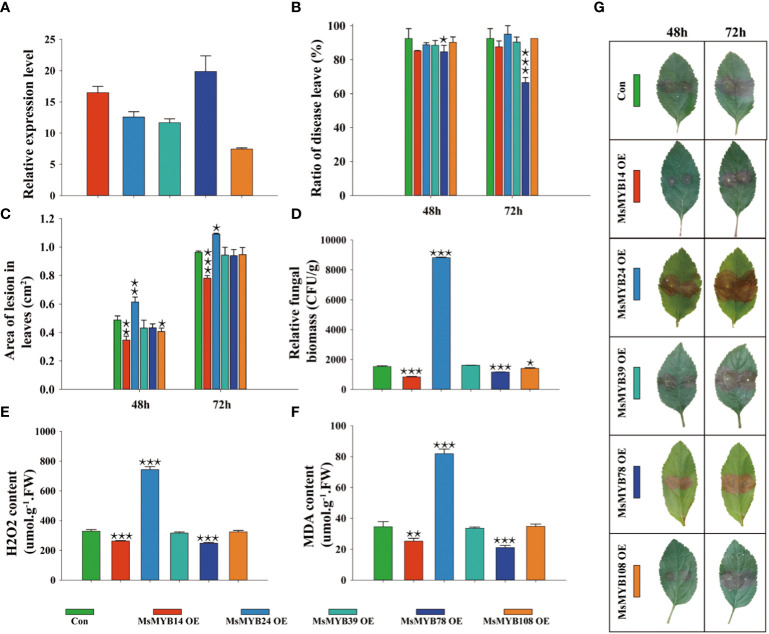
Functional identification of *MsMYB* family members in *Malus sieversii* in response to infection by *Valsa mali*. **(A)** Relative expression levels of *MsMYBs* in transiently transformed leaves determined by qRT-PCR at 72 h after infection by *V. mali*. The Valsa canker disease resistance of five *MsMYBs* is compared to that of the control. The lesion ratio **(B)** and area **(C)** were measured from detached leaves at 48 h and 72 h Each sample contained three or more independent biological replicates. One-way ANOVA was used for significances comparison with the control leaves (Con). **(D)** Relative fungal biomass of transiently transformed leaves of wild apple after 72 h of infection by *V. mali*. **(E)** H_2_O_2_ content in transiently transformed leaves of wild apple after 72 h of infection by *V. mali*. **(F)** MDA content in transiently transformed leaves of wild apple after 72 h of infection by *V. mali*. **(G)** The phenotype of transiently transformed leaves in wild apple changes following *V. mali* infection. One asterisk indicates a significant difference between treatment and control plants (p ≤ 0.05). Two asterisks indicate an extremely significant difference between treatment and control plants (p ≤ 0.01). Three asterisks indicate an extremely significant difference between treatment and control plants (p ≤ 0.001).

Five *MsMYBs* were cloned, and their expression levels in transiently transformed leaves were quantified by qRT-PCR. **Figure 7A** shows the lowest and highest relative expression level (7.45, *MsMYB108*; and 19.9, *MsMYB78*, respectively). This indicated that we successfully overexpressed *MsMYB14*, *MsMYB24*, *MsMYB39*, *MsMYB78*, and *MsMYB108* genes in wild apple leaves. The incidence of leaves overexpressing *MsMYB78* was 7.8% and 25.9% lower than that of the control at 48 h and 72 h, respectively. The leaves overexpressing *MsMYB78* reduced leaf incidence by 18.1% at 72 h compared to 48 h. This indicated that *MsMYB78* has a certain resistance phenotype. In contrast to the control plants, the remaining overexpressed leaves showed no significant difference in leaf incidence (**Figure 7B**). The leaves overexpressing the *MsMYB14* gene decreased the area of lesion leaves by 29%, which was statistically significant after 48 h. Additionally, it was decreased by 19% at 72 h, which was extremely significant. The lesion area in the leaves overexpressing the *MsMYB24* gene was increased by 26% and 13%, which were extremely significant and significant at 48 h and 72 h, respectively. When compared with the control plants, the lesion area of leaves overexpressing *MsMYB108* had been reduced by approximately 17% at 48 h. This indicates that *MsMYB14* was resistant, *MsMYB24* was susceptible, and *MsMYB108* had a slightly resistant phenotype (**Figure 7C**). **Figure 7D** shows a significant increase in fungal biomass in the *MsMYB24* overexpressed leaves when compared to the control leaves by approximately 600%, which was statistically significant. Compared to the control, the fungal biomass of *MsMYB14* and *MsMYB78* overexpressed leaves was reduced by 46% and 24%, respectively. The relative fungal biomass of the control was similar to that of *MsMYB39* and *MsMYB108* overexpressed leaves. **Figure 7D** supports these results and shows that *MsMYB78* has a disease resistant phenotype. Compared to the control, the leaves overexpressing *MsMYB24* had a 2.2-fold increase in H_2_O_2_ content, and the H_2_O_2_ content of the leaves overexpressing *MsMYB14* and *MsMYB78* decreased by 21% and 25%, respectively (**Figure 7E**). Moreover, compared with the control, the MDA content of the leaves overexpressing *MsMYB24* increased by 2.4 times and decreased by 27% and 39% in the leaves overexpressing *MsMYB14* and *MsMYB78*, respectively (**Figure 7F**). As shown in **Figures 7E and 7F**, *MsMYB14* and *MsMYB78* were resistant and *MsMYB24* was sensitive to *V. mali*. **Figure 7G** shows increased lesion area under *V. mali* infection at 48 h and 72 h on the leaves of wild apples. In summary, combined with the phenotypic traits shown in **Figure 7G**, we found that *MsMYB24* was sensitive to *V. mali*, *MsMYB14* and *MsMYB78* were resistance genes, and *MsMYB39* and *MsMYB108* had no function in response to *V. mali*.

## 4 Discussion

Recent research has revealed that wild apples (*Malus sieversii*) are the most primitive and chief ancestors of domesticated apples in the Tianshan Mountains of Central Asia and Western Europe (Cornille et al., 2014). The abundance of germplasm and genetic resources is conducive to molecular breeding of domesticated apples (Duan et al., 2016). There has been a drastic decrease in *M. sieversii* populations in Xinjiang as a result of apple canker disease caused by *V. mali*, a necrotrophic pathogen that has become widespread across Asia (Li X. et al., 2019). Therefore, it is imperative to explore the resistance gene resources in *M. sieversii* upon infection with *V. mali* to provide a basis for molecular breeding of apple trees and develop more effective strategies for preventing disease infection.

Our study focused on the MYB transcription factor family by conducting a genome-wide screening of the *M. sieversii* genome. Initially, 457 MsMYB transcription factor genes were identified in the genome of *M. sieversii*, including 128 genes of the *R2R3* type. Following *V. mali* infection, 27 *R2R3*-type *MsMYB* genes were found to be associated with the response to *V. mali* infection based on *M. sieversii* transcriptome data. To identify the genes involved in the response to infection with *V. mali*, we combined the transcriptome with the transient transformation method. In the present study, we found that wild apples can resist *V. mali* infection with *MsMYB14* and *MsMYB78*. In contrast, *MsMYB24* may play a negative role in the immunoregulation of wild apples.

According to the results of the present study, infection with *V. mali* significantly induced *MsMYB14* expression (**Figures 6A, B**). Compared with the control plants, leaves transiently overexpressing *MsMYB14* showed reduced lesion areas (**Figure 7C**), whereas the ratio of diseased leaves did not seem to differ. This suggests that *MsMYB14* enhances wild apple resistance by reducing *V. mali* propagation in leaves instead of invading the plant. According to Hurtado-Gaitán et al. (2021), *MYB14* enhanced the synthesis of resveratrol by down-regulating phosphoenolpyruvate carboxylase kinase in grapevines. Additionally, it may be directly associated with stilbene synthases (Holl et al., 2013; Fang et al., 2014; Wang et al., 2015) and stilbenoid pathways (Orduna et al., 2022), which are responsible for the production of resveratrol in grapevines. Plants produce stilbenes and resveratrol under biotic and abiotic stress as a defense mechanism. Duan et al. (2016) found that after a series of upstream signals (e.g., *RboH*-dependent oxidative burst, calcium influx, MAPK cascade, and jasmonate), *FLG22* induces the *MYB14* promoter, thus influencing resveratrol accumulation and resistance to pathogenic bacteria in plants. A recent study found that VqMAPKKK38, a member of the MAPK cascade, activates transcription factor MYB14 to positively regulate stilbene synthase transcription. It has been shown that the promoter of *VqMYB14* (*pVqMYB14*) is triggered by the elicitors *flg22* and harpin, respectively, and participates in both PAMP-triggered immunity and effector-triggered immunity (Luo et al., 2020). Using structure-activity relationships, Wang et al. (2020) showed that VqWRKY53 directly interacts with VqMYB14 and VqMYB15, enhancing stilbene synthesis. In addition, *MYB14* plays a role in the accumulation of flavonoids in *Marchantia polymorpha* (Hamashima et al., 2019) and conifers (Bedon et al., 2010), which were found to be defense phytoalexins in response to stress and infection. In contrast, no studies have been conducted to determine whether MYB14 is involved in the response to pathogenic fungi, specifically *V. mali.* Based on these findings, it is likely that *V. mali* infection induces *MsMYB14* expression, which in turn may result in increased biotic stress resistance through stilbenoid, resveratrol, or flavonoid accumulation.

However, leaves transiently overexpressing MsMYB78 showed a reduced ratio of diseased leaves (**Figure 7B**), whereas no obvious differences were observed in the lesion area compared to the control plants (**Figure 7C**). This suggests that MsMYB78 may improve wild apple resistance by reducing the incidence of *V. mali* invasion rather than by slowing the propagation of pathogens in leaves. CaMYB78 has been demonstrated to negatively regulate the anthocyanin biosynthetic pathway in chickpeas, culminating in increased resistance to *Fusarium oxysporum* (Shriti et al., 2022). It is likely that MYB78 is a broad-spectrum resistance gene that is responsive to pathogenic fungi such as *V. mali* and *F. oxysporum*.

Interestingly, *MsMYB24* played a negative role in the response to *V. mali* infection. The leaf lesion area (**Figure 7C**) and fungal biomass (**Figure 7D**) were significantly increased. Interaction between MYB24 and DELLA regulates filament elongation. Activation of *MYB24* encodes flavonol biosynthesis during Pollen Coat Patterning by regulating *FLS1* gene expression (Zhang X. et al., 2021) and phenylpropanoid biosynthesis during anther/pollen development by interacting with JAZ1/2 (Li et al., 2013). MYB24 is an influential regulator of jasmonate-mediated stamen development by interacting with bHLH TF (Song et al., 2011; Qi et al., 2015; Chen et al., 2016; Huang et al., 2017). Moreover, MYB24 plays a role in the growth of gynoecium, development of nectaries, and production of volatile sesquiterpenes, which may attract insects and/or repel pathogens (Reeves et al., 2012). As a result, MYB24 promotes plant reproduction by disrupting the balance between development and the stress response.

A synergistic effect of *MYB24* and *MYB108* on jasmonate-mediated stamen maturation has been reported in *Arabidopsis* (Mandaokar and Browse, 2009). The *MYB108* gene has been implicated in defense against *Verticillium dahliae* infection (Cheng et al., 2016) and has been shown to significantly increase anthocyanin biosynthesis (Khan et al., 2022) and regulate ABA-dependent wound-induced spreading cell death (Cui et al., 2013). Using *MsMYB108* overexpressing leaves, we found significant reductions in lesion areas after 48 h, but no difference in fungal biomass was observed. *MYB108* appears to participate in the disease response only at an early stage.

We identified 128 *MsMYB* genes of the *R2R3* type within the genome of *M. sieversii*. Based on the RNA-Seq results, we selected five TFs that may play an important role in the response to *V. mali.* In response to infection with *V. mali*, we characterized the functions of these five TFs in response to *V. mali* infection. We successfully identified two resistance genes and one sensitivity gene using RNA-Seq coupled with a molecular and physiological assay based on a transient genetic transformation platform. The methods used to identify resistance genes in response to biotic stress in the present study can be applied to a wide range of situations.

## Data availability statement

The datasets presented in this study can be found in online repositories. The names of the repository/repositories and accession number(s) can be found in the article/**Supplementary Material**.

## Author contributions

XW and DZ designed the research. YD, QY, and XW performed the research. QY, MZ, and XW analyzed the data and discussed the results. and QY, XW, and AW wrote the paper. All authors contributed to the article and approved the submitted version. The first two authors contributed equally.

## References

[B1] An J. P. Li R. Qu F. J. You C. X. Wang X. F. Hao Y. J. (2018). R2R3-MYB transcription factor *MdMYB23* is involved in the cold tolerance and proanthocyanidin accumulation in apple. Plant J. 96 (3), 562–577. doi: 10.1111/tpj.14050 30054966

[B2] Arce-Rodriguez M. L. Martinez O. Ochoa-Alejo N. (2021). Genome-wide identification and analysis of the MYB transcription factor gene family in chili pepper (*Capsicum* spp.). Int. J. Mol. Sci. 22 (5), 2229. doi: 10.3390/ijms22052229 33668082 PMC7956556

[B3] Bedon F. Bomal C. Caron S. Levasseur C. Boyle B. Mansfield S. D.. (2010). Subgroup 4 R2R3-MYBs in conifer trees: gene family expansion and contribution to the isoprenoid- and flavonoid-oriented responses. J. Exp. Bot. 61 (14), 3847–3864. doi: 10.1093/jxb/erq196 20732878 PMC2935864

[B4] Blanco E. Sabetta W. Danzi D. Negro D. Passeri V. Lisi A.. (2018). Isolation and characterization of the flavonol regulator CcMYB12 from the globe artichoke [*Cynara cardunculus* var. *scolymus* (L.) fiori]. Front. Plant Sci. 9. doi: 10.3389/fpls.2018.00941 PMC6042477 30026747

[B5] Cao Y. Li K. Li Y. Zhao X. Wang L. (2020). MYB transcription factors as regulators of secondary metabolism in plants. Biol. (Basel) 9 (3), 61. doi: 10.3390/biology9030061 PMC7150910 32213912

[B6] Cardenas-Hernandez H. Titaux-Delgado G. A. Castaneda-Ortiz E. J. Torres-Larios A. Brieba L. G. Del Rio-Portilla F.. (2021). Genome-wide and structural analysis of the *Myb-SHAQKYF* family in entamoeba histolytica. Biochim. Biophys. Acta Proteins Proteomics 1869 (4), 140601. doi: 10.1016/j.bbapap.2021.140601 33422669

[B7] Chen C. Chen H. Zhang Y. Thomas H. R. Frank M. H. He Y.. (2020). TBtools: An integrative toolkit developed for interactive analyses of big biological data. Mol. Plant 13 (8), 1194–1202. doi: 10.1016/j.molp.2020.06.009 32585190

[B8] Chen G. He W. Guo X. Pan J. (2021). Genome-wide identification, classification and expression analysis of the MYB transcription factor family in *Petunia* . Int. J. Mol. Sci. 22 (9), 4838. doi: 10.3390/ijms22094838 34063617 PMC8124715

[B9] Chen L. Hu B. Qin Y. Hu G. Zhao J. (2019). Advance of the negative regulation of anthocyanin biosynthesis by MYB transcription factors. Plant Physiol. Biochem. 136, 178–187. doi: 10.1016/j.plaphy.2019.01.024 30685697

[B10] Chen X. Huang H. Qi T. Liu B. Song S. (2016). New perspective of the bHLH-MYB complex in jasmonate-regulated plant fertility in *Arabidopsis* . Plant Signal. Behav. 11 (2), e1135280. doi: 10.1080/15592324.2015.1135280 26829586 PMC4883827

[B11] Chen X. Wang P. Gu M. Lin X. Hou B. Zheng Y.. (2021). R2R3-MYB transcription factor family in tea plant (*Camellia sinensis*): Genome-wide characterization, phylogeny, chromosome location, structure and expression patterns. Genomics 113 (3), 1565–1578. doi: 10.1016/j.ygeno.2021.03.033 33819564

[B12] Chen L. Yang H. Fang Y. Guo W. Chen H. Zhang X.. (2021). Overexpression of *GmMYB14* improves high-density yield and drought tolerance of soybean through regulating plant architecture mediated by the brassinosteroid pathway. Plant Biotechnol. J. 19 (4), 702–716. doi: 10.1111/pbi.1349615 33098207 PMC8051608

[B13] Cheng H. Q. Han L. B. Yang C. L. Wu X. M. Zhong N. Q. Wu J. H.. (2016). The cotton MYB108 forms a positive feedback regulation loop with CML11 and participates in the defense response against *Verticillium dahliae* infection. J. Exp. Bot. 67 (6), 1935–1950. doi: 10.1093/jxb/erw016 26873979 PMC4783372

[B14] Cornille A. Giraud T. Smulders M. J. Roldan-Ruiz I. Gladieux P. (2014). The domestication and evolutionary ecology of apples. Trends Genet. 30 (2), 57–65. doi: 10.1016/j.tig.2013.10.002 24290193

[B15] Cui F. Brosche M. Sipari N. Tang S. Overmyer K. (2013). Regulation of ABA dependent wound induced spreading cell death by MYB108. New Phytol. 200 (3), 634–640. doi: 10.1111/nph.12456 23952703

[B16] Daccord N. Celton J. M. Linsmith G. Becker C. Choisne N. Schijlen E.. (2017). High-quality *de novo* assembly of the apple genome and methylome dynamics of early fruit development. Nat. Genet. 49 (7), 1099–1106. doi: 10.1038/ng.3886 28581499

[B17] Duan D. Fischer S. Merz P. Bogs J. Riemann M. Nick P. (2016). An ancestral allele of grapevine transcription factor MYB14 promotes plant defence. J. Exp. Bot. 67 (6), 1795–1804. doi: 10.1093/jxb/erv569 26842984 PMC4783363

[B18] Dubos C. Stracke R. Grotewold E. Weisshaar B. Martin C. Lepiniec L. (2010). MYB transcription factors in *Arabidopsis* . Trends Plant Sci. 15 (10), 573–581. doi: 10.1016/j.tplants.2010.06.005 20674465

[B19] Faize M. Faize L. Alburquerque N. Venisse J. S. Burgos L. (2020). Hydrogen peroxide generated by over-expression of cytosolic superoxide dismutase in transgenic plums enhances bacterial canker resistance and modulates plant defence responses. Mol. Biol. Rep. 47 (8), 5889–5901. doi: 10.1007/s11033-020-05660-8 32661871

[B20] Fang L. Hou Y. Wang L. Xin H. Wang N. Li S. (2014). Myb14, a direct activator of STS, is associated with resveratrol content variation in berry skin in two grape cultivars. Plant Cell Rep. 33 (10), 1629–1640. doi: 10.1007/s00299-014-1642-3 24948530

[B21] Hamashima N. Xie X. Hikawa M. Suzuki T. Kodama Y. (2019). A gain-of-function T-DNA insertion mutant of marchantia polymorpha hyper-accumulates flavonoid riccionidin a. Plant Biotechnol. (Tokyo) 36 (3), 201–204. doi: 10.5511/plantbiotechnology.19.0722a 31768123 PMC6854341

[B22] Holl J. Vannozzi A. Czemmel S. D’Onofrio C. Walker A. R. Rausch T.. (2013). The R2R3-MYB transcription factors *MYB14* and *MYB15* regulate stilbene biosynthesis in *Vitis vinifera* . Plant Cell 25 (10), 4135–4149. doi: 10.1105/tpc.113.117127 24151295 PMC3877794

[B23] Huang H. Gao H. Liu B. Qi T. Tong J. Xiao L.. (2017). *Arabidopsis MYB24* regulates jasmonate-mediated stamen development. Front. Plant Sci. 8, 1525. doi: 10.3389/fpls.2017.01525 28928760 PMC5591944

[B24] Hurtado-Gaitán E. Sellés-Marchart S. Hartwell J. Martínez-Esteso M. J. Bru-Martínez R. (2021). Down-regulation of phosphoenolpyruvate carboxylase kinase in grapevine cell cultures and leaves is linked to enhanced resveratrol biosynthesis. Biomolecules 11 (11), 1641. doi: 10.3390/biom11111641 34827639 PMC8615455

[B25] IH O. EP R. (1999). The myb gene family in cell growth, differentiation and apoptosis. Oncogene 118 (19), 3017–3033. doi: 10.1038/sj.onc.1202839 10378697

[B26] Jin H. Martin C. (1999). Multifunctionality and diversity within the plant MYB-gene family. Plant Mol. Biol. 41 (5), 577–585. doi: 10.1023/a:1006319732410 10645718

[B27] Kanei-Ishii C. Sarai A. Sawazaki T. Nakagoshi H. He D. N. Ogata K.. (1990). The tryptophan cluster: a hypothetical structure of the DNA-binding domain of the myb protooncogene product. J. Biol. Chem. 265 (32), 19990–19995. doi: 10.1016/s0021-9258(17)45472-x 2246275

[B28] Khan I. A. Cao K. Guo J. Li Y. Wang Q. Yang X.. (2022). Identification of key gene networks controlling anthocyanin biosynthesis in peach flower. Plant Sci. 316, 111151. doi: 10.1016/j.plantsci.2021.111151 35151460

[B29] Kranz H. Scholz K. Weisshaar B. (2000). C-MYB oncogene-like genes encoding three MYB repeats occur in all major plant lineages. Plant J. 21 (2), 231–235. doi: 10.1046/j.1365-313x.2000.00666.x 10743663

[B30] Lee D. H. Lee S. W. Choi K. H. Kim D. A. Uhm J. Y. (2006). Survey on the occurrence of apple diseases in Korea from 1992 to 2000. Plant Pathol. J. 22 (4), 375–380. doi: 10.5423/ppj.2006.22.4.375

[B31] Li X. Guo C. Ahmad S. Wang Q. Yu J. Liu C.. (2019). Systematic analysis of *MYB* family genes in potato and their multiple roles in development and stress responses. Biomolecules 9 (8), 317. doi: 10.3390/biom9080317 31366107 PMC6723670

[B32] Li J. Han G. Sun C. Sui N. (2019). Research advances of MYB transcription factors in plant stress resistance and breeding. Plant Signal. Behav. 14 (8), 1613131. doi: 10.1080/15592324.2019.1613131 31084451 PMC6619938

[B33] Li Y. Jiang J. Du M. L. Li L. Wang X. L. Li X. B. (2013). A cotton gene encoding MYB-like transcription factor is specifically expressed in pollen and is involved in regulation of late anther/pollen development. Plant Cell Physiol. 54 (6), 893–906. doi: 10.1093/pcp/pct038 23447105

[B34] Li L. Zhang S. Wang B. (2021). Apple leaf disease identification with a small and imbalanced dataset based on lightweight convolutional networks. Sens. (Basel) 22 (1), 173. doi: 10.3390/s22010173 PMC8749501 35009716

[B35] Liang X. Zhang R. Gleason M. L. Sun G. (2022). Sustainable apple disease management in China: Challenges and future directions for a transforming industry. Plant Dis. 106 (3), 786–799. doi: 10.1094/PDIS-06-21-1190-FE 34698518

[B36] Liu X. Li X. Wen X. Zhang Y. Ding Y. Zhang Y.. (2021). PacBio full-length transcriptome of wild apple (*Malus sieversii*) provides insights into canker disease dynamic response. BMC Genom. 22 (1), 52. doi: 10.1186/s12864-021-07366-y PMC7809858 33446096

[B37] Livak K. J. Schmittgen T. D. (2001). Analysis of relative gene expression data using real-time quantitative PCR and the 2(-delta delta C(T)) method. Methods 25 (4), 402–408. doi: 10.1006/meth.2001.1262 11846609

[B38] Lu S. X. Knowles S. M. Andronis C. Ong M. S. Tobin E. M. (2009). CIRCADIAN CLOCK ASSOCIATED1 and LATE ELONGATED HYPOCOTYL function synergistically in the circadian clock of *Arabidopsis* . Plant Physiol. 150 (2), 834–843. doi: 10.1104/pp.108.133272 19218364 PMC2689956

[B39] Luo Y. Wang Q. Bai R. Li R. Chen L. Xu Y.. (2020). The effect of transcription factor *MYB14* on defense mechanisms in *Vitis quinquangularis-pingyi* . Int. J. Mol. Sci. 21 (3), 706. doi: 10.3390/ijms21030706 31973146 PMC7036875

[B40] Mandaokar A. Browse J. (2009). *MYB108* acts together with *MYB24* to regulate jasmonate-mediated stamen maturation in arabidopsis. Plant Physiol. 149 (2), 851–862. doi: 10.1104/pp.108.132597 19091873 PMC2633834

[B41] Mmadi M. A. Dossa K. Wang L. Zhou R. Wang Y. Cisse N.. (2017). Functional characterization of the versatile *MYB* gene family uncovered their important roles in plant development and responses to drought and waterlogging in *Sesame* . Genes (Basel) 8 (12), 362. doi: 10.3390/genes8120362 29231869 PMC5748680

[B42] Ogata K. Hojo H. Aimoto S. Nakai T. Nakamura H. Sarai A.. (1992). Solution structure of a DNA-binding unit of myb: A helix-turn-helix-related motif with conserved tryptophans forming a hydrophobic core. Proc. Natl. Acad. Sci. 89 (14), 6428–6432. doi: 10.1073/pnas.89.14.6428 1631139 PMC49514

[B43] Ogata K. Kanei-Ishii C. Sasaki M. Hatanaka H. Nagadoi A. Enari M.. (1996). The cavity in the hydrophobic core of myb DNA-binding domain is reserved for DNA recognition and trans-activation. Nat. Struct. Biol. 3 (2), 178–187. doi: 10.1038/nsb0296-178 8564545

[B44] Orduna L. Li M. Navarro-Paya D. Zhang C. Santiago A. Romero P.. (2022). Direct regulation of shikimate, early phenylpropanoid, and stilbenoid pathways by subgroup 2 R2R3-MYBs in grapevine. Plant J. 110 (2), 529–547. doi: 10.1111/tpj.15686 35092714

[B45] Paz-Ares J. Ghosal D. Wienand U. A.Petersont P. Saedler H. (1987). The regulatory cl locus of zea mays encodes a protein with homology to myb proto-oncogene products and with structural similarities to transcriptional activators. EMBO J. 6 (12), 3553–3558. doi: 10.1002/j.1460-2075 3428265 PMC553820

[B46] Pucker B. Pandey A. Weisshaar B. Stracke R. (2020). The *R2R3-MYB* gene family in banana (*Musa acuminata*): Genome-wide identification, classification and expression patterns. PloS One 15 (10), e0239275. doi: 10.1371/journal.pone.0239275 33021974 PMC7537896

[B47] Qi T. Huang H. Song S. Xie D. (2015). Regulation of jasmonate-mediated stamen development and seed production by a bHLH-MYB complex in *Arabidopsis* . Plant Cell 27 (6), 1620–1633. doi: 10.1105/tpc.15.00116 26002869 PMC4498206

[B48] Reeves P. H. Ellis C. M. Ploense S. E. Wu M. F. Yadav V. Tholl D.. (2012). A regulatory network for coordinated flower maturation. PLoS Genet. 8 (2), e1002506. doi: 10.1371/journal.pgen.1002506 22346763 PMC3276552

[B49] Riechmann J. L. Heard J. Martin G. Reuber L. Jiang C. Keddie J.. (2000). *Arabidopsis* transcription factors: Genome-wide comparative analysis among eukaryotes. Science 290 (5499), 2105–2110. doi: 10.1126/science.290.5499.2105 11118137

[B50] Romero I. Fuertes A. Benito M. J. Malpica J. M. Leyva A. Paz-Ares J. (1998). More than *80R2R3-MYB* regulatory genes in the genome of *Arabidopsis thaliana* . Plant J. 14 (3), 273–283. doi: 10.1046/j.1365-313x.1998.00113.x 9628022

[B51] Rosinski J. A. Atchley W. R. (1998). Molecular evolution of the myb family of transcription factors: Evidence for polyphyletic origin. J. Mol. Evol. 46 (1), 74–83. doi: 10.1007/pl00006285 9419227

[B52] Shriti S. Paul S. Das S. (2022). Overexpression of CaMYB78 transcription factor enhances resistance response in chickpea against fusarium oxysporum and negatively regulates anthocyanin biosynthetic pathway. Protoplasma 260 (2), 589–605. doi: 10.1007/s00709-022-01797-4 35947211

[B53] Song S. Qi T. Huang H. Ren Q. Wu D. Chang C.. (2011). The jasmonate-ZIM domain proteins interact with the R2R3-MYB transcription factors MYB21 and MYB24 to affect jasmonate-regulated stamen development in *Arabidopsis* . Plant Cell 23 (3), 1000–1013. doi: 10.1105/tpc.111.083089 21447791 PMC3082250

[B54] Song X. Yang Q. Liu Y. Li J. Chang X. Xian L.. (2021). Genome-wide identification of pistacia *R2R3-MYB* gene family and function characterization of PcMYB113 during autumn leaf coloration in *Pistacia chinensis* . Int. J. Biol. Macromol. 192, 16–27. doi: 10.1016/j.ijbiomac.2021.09.092 34555399

[B55] Stracke R. Werber M. Weisshaar B. (2001). The *R2R3-MYB* gene family in *Arabidopsis thaliana* . Curr. Opin. Plant Biol. 4 (5), 447–456. doi: 10.1016/s1369-5266(00)00199-0 11597504

[B56] Sun W. Ma Z. Chen H. Liu M. (2019). *MYB* gene family in potato (*Solanum tuberosum l.*): Genome-wide identification of hormone-responsive reveals their potential functions in growth and development. Int. J. Mol. Sci. 20 (19), 4847. doi: 10.3390/ijms20194847 31569557 PMC6801432

[B57] Tuan P. A. Bai S. Yaegaki H. Tamura T. Hihara S. Moriguchi T.. (2015). The crucial role of *PpMYB10.1* in anthocyanin accumulation in peach and relationships between its allelic type and skin color phenotype. BMC Plant Biol. 15, 280. doi: 10.1186/s12870-015-0664-5 26582106 PMC4652394

[B58] Wang D. Jiang C. Liu W. Wang Y. (2020). The WRKY53 transcription factor enhances stilbene synthesis and disease resistance by interacting with MYB14 and MYB15 in Chinese wild grape. J. Exp. Bot. 71 (10), 3211–3226. doi: 10.1093/jxb/eraa097 32080737

[B59] Wang J. F. Ma L. Xi H. F. Wang L. J. Li S. H. (2015). Resveratrol synthesis under natural conditions and after UV-c irradiation in berry skin is associated with berry development stages in ‘Beihong’ (*V. vinifera*×*V. amurensis*). Food Chem. 168, 430–438. doi: 10.1016/j.foodchem.2014.07.025 25172731

[B60] Wang X. Zang R. Yin Z. Kang Z. Huang L. (2014). Delimiting cryptic pathogen species causing apple valsa canker with multilocus data. Ecol. Evol. 4 (8), 1369–1380. doi: 10.1002/ece3.1030 24834333 PMC4020696

[B61] Wen X. Ding Y. Tan Z. Wang J. Zhang D. Wang Y. (2020). Identification and characterization of cadmium stress-related LncRNAs from *Betula platyphylla* . Plant Sci. 299, 110601. doi: 10.1016/j.plantsci.2020.110601 32900439

[B62] Xie Y. Chen P. Yan Y. Bao C. Li X. Wang L.. (2018). An atypical R2R3 MYB transcription factor increases cold hardiness by CBF-dependent and CBF-independent pathways in apple. New Phytol. 218 (1), 201–218. doi: 10.1111/nph.14952 29266327

[B63] Xing G. Li J. Li W. Lam S. M. Yuan H. Shui G.. (2021). *AP2*/*ERF* and *R2R3-MYB* family transcription factors: potential associations between temperature stress and lipid metabolism in *Auxenochlorella protothecoides* . Biotechnol. Biofuels 14 (1), 22. doi: 10.1186/s13068-021-01881-6 33451355 PMC7811268

[B64] Yanhui C. Xiaoyuan Y. Kun H. Meihua L. Jigang L. Zhaofeng G.. (2006). The MYB transcription factor superfamily of *Arabidopsis*: expression analysis and phylogenetic comparison with the rice *MYB* family. Plant Mol. Biol. 60 (1), 107–124. doi: 10.1007/s11103-005-2910-y 16463103

[B65] Yuan Y. Yang X. Feng M. Ding H. Khan M. T. Zhang J.. (2021). Genome-wide analysis of R2R3-MYB transcription factors family in the autopolyploid saccharum spontaneum: an exploration of dominance expression and stress response. BMC Genom. 22 (1), 622. doi: 10.1186/s12864-021-07689-w PMC8371785 34404342

[B66] Zhang X. He Y. Li L. Liu H. Hong G. (2021). Involvement of the R2R3-MYB transcription factor MYB21 and its homologs in regulating flavonol accumulation in *Arabidopsis stamen* . J. Exp. Bot. 72 (12), 4319–4332. doi: 10.1093/jxb/erab156 33831169 PMC8163065

[B67] Zhang Q. Wang L. Wang Z. Zhang R. Liu P. Liu M.. (2021). The regulation of cell wall lignification and lignin biosynthesis during pigmentation of winter jujube. Hortic. Res. 8 (1), 238. doi: 10.1038/s41438-021-00670-4 34719675 PMC8558337

[B68] Zhang L. Zhao G. Jia J. Liu X. Kong X. (2012). Molecular characterization of 60 isolated wheat *MYB* genes and analysis of their expression during abiotic stress. J. Exp. Bot. 63 (1), 203–214. doi: 10.1093/jxb/err264 21934119 PMC3245462

[B69] Zhou F. Chen Y. Wu H. Yin T. (2021). Genome-wide comparative analysis of *R2R3 MYB* gene family in *Populus* and *Salix* and identification of male flower bud development-related genes. Front. Plant Sci. 12. doi: 10.3389/fpls.2021.721558 PMC8477045 34594352

